# Use of ChatGPT to Explore Gender and Geographic Disparities in Scientific Peer Review

**DOI:** 10.2196/57667

**Published:** 2024-12-09

**Authors:** Paul Sebo

**Affiliations:** 1 University of Geneva Geneva Switzerland

**Keywords:** Africa, artificial intelligence, discrimination, peer review, sentiment analysis, ChatGPT, disparity, gender, geographic, global south, inequality, woman, assessment, researcher, communication, consultation, gender bias

## Abstract

**Background:**

In the realm of scientific research, peer review serves as a cornerstone for ensuring the quality and integrity of scholarly papers. Recent trends in promoting transparency and accountability has led some journals to publish peer-review reports alongside papers.

**Objective:**

ChatGPT-4 (OpenAI) was used to quantitatively assess sentiment and politeness in peer-review reports from high-impact medical journals. The objective was to explore gender and geographical disparities to enhance inclusivity within the peer-review process.

**Methods:**

All 9 general medical journals with an impact factor >2 that publish peer-review reports were identified. A total of 12 research papers per journal were randomly selected, all published in 2023. The names of the first and last authors along with the first author’s country of affiliation were collected, and the gender of both the first and last authors was determined. For each review, ChatGPT-4 was asked to evaluate the “sentiment score,” ranging from –100 (negative) to 0 (neutral) to +100 (positive), and the “politeness score,” ranging from –100 (rude) to 0 (neutral) to +100 (polite). The measurements were repeated 5 times and the minimum and maximum values were removed. The mean sentiment and politeness scores for each review were computed and then summarized using the median and interquartile range. Statistical analyses included Wilcoxon rank-sum tests, Kruskal-Wallis rank tests, and negative binomial regressions.

**Results:**

Analysis of 291 peer-review reports corresponding to 108 papers unveiled notable regional disparities. Papers from the Middle East, Latin America, or Africa exhibited lower sentiment and politeness scores compared to those from North America, Europe, or Pacific and Asia (sentiment scores: 27 vs 60 and 62 respectively; politeness scores: 43.5 vs 67 and 65 respectively, adjusted *P*=.02). No significant differences based on authors’ gender were observed (all *P*>.05).

**Conclusions:**

Notable regional disparities were found, with papers from the Middle East, Latin America, and Africa demonstrating significantly lower scores, while no discernible differences were observed based on authors’ gender. The absence of gender-based differences suggests that gender biases may not manifest as prominently as other forms of bias within the context of peer review. The study underscores the need for targeted interventions to address regional disparities in peer review and advocates for ongoing efforts to promote equity and inclusivity in scholarly communication.

## Introduction

The peer-review process plays a pivotal role in validating the quality and integrity of scholarly papers. With an increasing emphasis on transparency and accountability, some journals adopted the practice of publishing peer-review reports alongside papers.

Sentiment analysis, the identification and categorization of opinions, attitudes, and emotions conveyed in text data as positive, negative, or neutral [1], finds ChatGPT (OpenAI) standing out with unique advantages compared to “traditional methods” [1-5]. Unlike lexicon-based approaches that may overlook nuanced expressions, ChatGPT understands natural language, capturing subtle nuances and context-specific sentiments. Unlike machine learning methods requiring labeled datasets, ChatGPT adapts to diverse domains without explicit training, reducing costs and time. Its humanlike responses facilitate intuitive sentiment interpretation. Additionally, artificial intelligence (AI)–driven sentiment analysis, including ChatGPT, ensures efficient, scalable (able to accommodate increasing data volumes without significant performance degradation), consistent, objective (devoid of human biases and preconceptions), and adaptable analyses. ChatGPT showed promise for sentiment analysis in several recent studies [6-10].

Verharen [11] showed that ChatGPT was accurate in determining sentiment and politeness scores in peer reviews of scientific papers. The study also showed gender inequalities, with woman authors receiving less polite reviews than men. The study, limited to papers from a single journal (*Nature Communications*), aligns with the broader issue of gender discrimination in academic medicine [12-17]. While sentiment and politeness metrics may not directly measure bias, they serve as useful proxies for identifying potential biases in peer review. Biased reviewers may exhibit tendencies toward overly positive or negative sentiments, as well as varying levels of politeness toward authors based on factors such as gender or geographic region.

Building upon Verharen’s [11] work, ChatGPT-4 was used to quantitatively assess sentiment and politeness in peer-review reports from 9 high-impact medical journals, exploring gender and geographical disparities to enhance inclusivity within the peer review process. By leveraging the capabilities of AI, this study sheds light on the potential of AI technologies to mitigate biases and promote fairness in scholarly communication.

## Methods

### Selection of Journals, Papers, and Peer-Review Reports

The Clarivate and ASAPbio websites were searched to identify all 9 general medical journals with a journal citation reports impact factor >2 that publish peer-review reports (Table 1). None of these journals uses double-blind peer review. Using simple randomization, which involves randomly selecting papers using a random number generator, 12 research papers per journal were selected, all published in 2023, and all peer-review reports from the initial round for these 108 papers were retrieved. This sample size was chosen to ensure robust and manageable analysis.

**Table 1:** List of high-impact general medical journals included in this cross-sectional study using ChatGPT-4 to quantitatively assess sentiment and politeness in 291 peer-review reports for 108 papers published in 2023, as well as number of papers and reviews per journal, and median sentiment and politeness scores per journal.

Journal	2022 impact factor, n	Papers (n=108), n (%)	Reviews (n=291), n (%)	Sentiment score, median (IQR)	Politeness score, median (IQR)
*BMJ*	107.7	12 (11.1)	51 (17.5)	68 (53-78)	73 (60-80)
*PLOS Medicine*	15.8	12 (11.1)	41 (14.1)	60 (50-73)	70 (62-78)
*BMC Medicine*	9.3	12 (11.1)	29 (10.0)	63 (48-70)	73 (60-78)
*Journal of Clinical Medicine*	3.9	12 (11.1)	31 (10.7)	43 (17-70)	50 (25-67)
*Diagnostics*	3.6	12 (11.1)	28 (9.6)	37 (3-60)	57 (28.5-68.5)
*Journal of Personalized Medicine*	3.4	12 (11.1)	27 (9.3)	57 (17-70)	68 (27-82)
*BMJ Open*	2.9	12 (11.1)	27 (9.3)	63 (40-72)	60 (45-70)
*BMC Primary Care*	2.9	12 (11.1)	25 (8.6)	57 (10-67)	55 (30-70)
*Medicina*	2.6	12 (11.1)	32 (11.0)	48.5 (20-65)	57 (43.5-66)

### Data Collection and Gender Determination

The names of the first and last authors along with the first author’s country of affiliation were collected, and the gender of both the first and last authors was determined. The authors’ genders were categorized in 2 steps. The gender was determined based on names alone, classifying names as man or woman accordingly. For authors whose gender could not be inferred from their names, professional networks and university websites were searched for photos or text containing gender-specific pronouns. This method enabled to assign genders to all authors. A gender detection tool (Gender API; Markus Perl IT Solutions) was also used to confirm the classifications [18]. Gender API demonstrated high accuracy in previous studies [19]. Both approaches yielded similar results, with high agreement between them (first authors: percentage agreement=0.9725, Cohen κ=0.9450; last authors: percentage agreement=0.9691, Cohen κ=0.9295).

### Sentiment and Politeness Scores

For each review, ChatGPT-4 was asked to evaluate the “sentiment score,” ranging from –100 (negative) to +100 (positive), and the “politeness score,” ranging from –100 (rude) to +100 (polite). The measurements were repeated 5 times and the minimum and maximum values were removed. The sentiment score measures how favorable the review is, and the politeness score how polite a review’s language is. The same prompt as Verharen [11] was used:

Below you will find a scientific peer review. Can you score this peer review on the sentiment, on a scale from –100 (negative) to 0 (neutral) to 100 (positive), and politeness of language use, on a scale of –100 (rude) to 0 (neutral) to 100 (polite)?

All the data were collected in January 2024. The data associated with this paper are available in the Open Science Framework [20]. The database is provided as a “dta” file for use with Stata. Multimedia Appendix 1 provides a detailed list and description of the variables included in the Stata file.

### Statistical Analyses

The mean sentiment and politeness scores for each review were computed and rounded to the nearest whole number. These scores were then summarized using the median and IQR, both overall and stratified by journal, gender, and affiliation. Affiliation countries were categorized into 3 regions (North America, Europe, or Pacific; Asia; and Latin America, Middle East, or Africa), following prior research [21]. Comparisons were conducted using Wilcoxon rank-sum tests (for gender) and Kruskal-Wallis rank tests (for affiliation). Negative binomial regressions were performed, adjusting for journal, affiliation, and intracluster correlation within papers [22,23]. To accommodate the requirement of nonnegative outcome variables in negative binomial regressions, 100 were added to the sentiment and politeness scores, resulting in a scale from 0 to 200. Quadratic weighted agreement coefficients were calculated to assess the agreement between the 3 sentiment and politeness score measurements. Fleiss κ was used instead of Cohen κ due to the presence of more than 2 measurements [24]. All analyses were conducted using Stata (version 15.1; StataCorp).

### Ethical Considerations

Since this study did not involve the collection of personal health-related data, it did not require ethical review, according to the current Swiss law.

## Results

There were 291 reviews for the 108 papers selected for the study (Multimedia Appendix 2). Men were the first and last authors of 61 (56.5%) and 75 (69.4%) papers, respectively. The 5 most represented countries of affiliation were the United Kingdom (n=14), Germany (n=13), the United States (n=10), China (n=9), and Italy (n=6). The 3 main regions of affiliation were Western Europe (n=56), Asia (n=16), and North America (n=15).

Overall, the median sentiment and politeness scores were 58 (IQR 30-72; range –70 to 90) and 63 (IQR 47-75; range –73 to 92), respectively, but there were notable variations by the journal (Table 1). The 3 journals with the highest impact factor tended to have higher sentiment and politeness scores. There was no significant difference in scores between men and women (all *P*>.05; Table 2). The results were almost identical when using Gender API to determine gender (first authors: median sentiment or politeness scores=58, IQR 37-72 and 65, IQR 47-77 for women, 57.5, IQR 27-70 and 63, IQR 47-73 for men; last authors: median sentiment or politeness scores=55, IQR 33-68 and 63, IQR 50-75 for women, 58, IQR 27-72 and 63, IQR 45-75 for men). By contrast, papers authored by scholars from countries in the Middle East, Latin America, or Africa exhibited significantly lower sentiment and politeness scores compared to those from the other 2 regions, with differences exceeding 30 and 20 points in absolute value, respectively (adjusted *P*=.02; Table 2).

In light of the *BMJ*’s notable impact factor compared to the other journals in the study, additional analyses were conducted by excluding the 51 peer-review reports for *BMJ*. The results remained consistent (Multimedia Appendix 3). The interrater agreement between the 3 measurements was high (sentiment scores: percentage agreement=0.9958, 95% CI 0.9954-0.9962; Fleiss κ=0.9496, 95% CI 0.9395-0.9598; politeness scores: percentage agreement=0.9962, 95% CI 0.9958-0.9966; Fleiss κ=0.9463, 95% CI 0.9316-0.9610; all *P*<.001).

**Table 2:** Associations between sentiment or politeness scores and first or last authors’ gender and first authors’ affiliation in this cross-sectional study using ChatGPT-4 to quantitatively assess sentiment and politeness in 291 peer-review reports for 108 papers published in 2023 in 9 high-impact general medical journals.

Variable			Papers (n=108), n (%)		Reviews (n=291), n (%)		Sentiment score, median (IQR)		Crude *P* value^a^			Adjusted *P* value^b^			Politeness score, median (IQR)			Crude *P* value^a^			Adjusted *P* value^b^	
**First authors’ gender**										.49			.48						.37			.68
	Woman	47 (43.5)		127 (43.6)		58 (33-72)								65 (47-77)								
	Man	61 (56.5)		164 (56.4)		57.5 (27-70)								63 (48.5-73)								
**Last authors’ gender**										.52			.88						.74			.86
	Woman	33 (30.6)		91 (31.3)		57 (33-68)								63 (50-75)								
	Man	75 (69.4)		200 (68.7)		60 (27.5-72)								63 (45-75)								
**First authors’ affiliation**										.001			.02^c^						<.001			.02^d^
	North America, Europe, and Pacific	82 (75.9)		220 (75.6)		60 (33-72)								67 (53-77)								
	Asia	16 (14.8)		43 (14.8)		62 (40-70)								65 (50-73)								
	Middle East, Latin America, and Africa	10 (9.3)		28 (9.6)		27 (–3 to 55)								43.5 (17.5-57)								

^a^Wilcoxon rank-sum test (for gender) and Kruskal-Wallis equality-of-populations rank test (for affiliation).

^b^Multivariable negative binomial regression, adjusted for journal, affiliation, and intracluster correlation within papers (for first or last authors’ gender), and adjusted for journal and intracluster correlation within papers (for first authors’ affiliation).

^c^Incidence rate ratio: Asia versus Middle East, Latin America, and Africa: 1.27 (95% CI 1.06-1.51); North America, Europe, and Pacific versus Middle East, Latin America, and Africa: 1.23 (95% CI 1.02-1.47).

^d^Incidence rate ratio: Asia versus Middle East, Latin America, and Africa: 1.30 (95% CI 1.07-1.57); North America, Europe, and Pacific versus Middle East, Latin America, and Africa: 1.27 (95% CI 1.04-1.54).

## Discussion

### Principal Findings

ChatGPT-4 was used to analyze sentiment and politeness in 291 peer-review reports from 9 general medical journals. The study unveiled notable regional disparities, with papers from the Middle East, Latin America, or Africa demonstrating significantly lower scores, while no discernible differences were observed based on the authors’ gender.

### Comparison With Existing Literature

The gender disparities experienced by women in academic medicine are widely recognized [12-17]. Consequently, significant gender discrepancies were anticipated in this study to the detriment of women. The absence of discernible differences between genders contrasts with Verharen’s [11] findings, where woman first authors typically received less polite reviews compared to men. Importantly, the study focused on a different discipline (neuroscience) and was confined to a single journal (*Nature Communications*).

The regional disparities highlighted in the study resonate with prior research illustrating the challenges faced by researchers, particularly those from countries in the Global South [21,25]. These findings align with those of earlier studies [26-28] and must be understood within broader sociocultural, economic, and institutional contexts. Factors such as limited funding opportunities, language barriers, and cultural differences may contribute to biases against authors from underrepresented regions. Additionally, institutional biases within academic publishing systems may further exacerbate these discrepancies, highlighting the need for interventions to foster equity and inclusivity in scholarly discourse.

The observed disparities across geographic regions, contrasted with the absence of significant differences based on gender, could indicate that within the context of peer review, gender biases may not manifest as prominently or uniformly as other forms of bias, such as those influenced by geographic factors. In addition, existing diversity or inclusion initiatives within the scholarly community may have been more effective in mitigating gender disparities compared to other forms of bias.

### Strengths and Limitations

This study built on the findings of Verharen [11], who demonstrated through several validation methods the accuracy of ChatGPT in estimating sentiment and politeness in peer-review reports, surpassing that of human evaluation and traditional lexicon-based language models. This study has several limitations, including its exclusive focus on high-impact general medical journals, reliance on binary gender determination without consideration for nonbinary or transgender identities, and uncertainty about gender or geographic distributions of rejected papers. Future research should explore alternative approaches for gender determination such as self-identification for accurately capturing gender diversity. In addition, the sample size (108 papers and 291 peer-review reports) is smaller than that of prior research by Verharen [11], potentially limiting the generalizability of the findings. The author also acknowledges the challenges associated with using sentiment or politeness metrics to capture the nuanced biases inherent in peer review, and the dependence on algorithms like ChatGPT may introduce potential inaccuracies. Furthermore, manual scoring was not conducted for comparison. Finally, the observed association between sentiment and politeness metrics and affiliation regions suggests an alternative explanation: the possibility of higher scientific merit in papers from certain authors. The methodology lacks the capability to discern between the 2 hypotheses.

### Conclusions

ChatGPT-4 demonstrated effectiveness in this study by consistently evaluating sentiment and politeness in peer-review reports. The study underscores the need for targeted interventions to address regional disparities in peer review and advocates for ongoing efforts to promote equity and inclusivity in scholarly communication.

### Data Availability

The datasets generated and analyzed during this study are available in the Open Science Framework (OSF) repository [20].

## Appendix

Multimedia Appendix 1 List and description of variables available in the Stata file uploaded to the Open Science Framework (OSF).

Multimedia Appendix 2 List of papers and reviews.

Multimedia Appendix 3 Associations between sentiment or politeness scores and first or last authors’ gender and first authors’ affiliation in this cross-sectional study using ChatGPT-4, to quantitatively assess sentiment and politeness in 240 peer review reports for 96 articles published in 2023 (same data as in Table 2 but without taking into account the 51 peer-review reports for the 12 articles published in BMJ).

## Abbreviations

AI: artificial intelligence
