# Supplementary material for: Use of ChatGPT to Explore Gender and Geographic Disparities in Scientific Peer Review
Source: J Med Internet Res. 2024 Dec 9;26:e57667. doi: 10.2196/57667 (PMC11667125; doi:10.2196/57667)
Supplement: Multimedia Appendix 1 [file jmir_v26i1e57667_app1.docx]

**‘journal’**:

Name of the journal in which the article was published (e.g. ‘BMJ’)

**‘article’**:

Number of the article published in the journal (e.g. article ‘101’ published in ‘BMJ’)

**‘review’**:

Number of the peer review (e.g. article ‘101’ published in ‘BMJ’ has undergone four peer reviews, i.e. reviews ‘1’, ‘2’, ‘3’, and ‘4’)

**‘gender_first’** (F=female or M=male):

Gender of the first author (e.g. ‘F’ for the gender of the first author of article ‘101’)

**‘gender_last’** (F=female or M=male):

Gender of the last author (e.g. ‘F’ for the gender of the last author of article ‘101’)

**‘country’**:

Country of affiliation of the first author (for example ‘USA’ for article ‘101’)

**‘region’** (Africa, Asia, EE=East Europe, ME=Middle East, NA=North America, P=Pacific, SA=South America, WE=Western Europe):

Region of affiliation of the first author (e.g. ‘NA’ for article ‘101’)

**‘S1’**:

Sentiment score for the first measurement

**‘S2’**:

Sentiment score for the second measurement

**‘S3’**:

Sentiment score for the third measurement

**‘P1’**:

Politeness score for the first measurement

**‘P2’**:

Politeness score for the second measurement

**‘P3’**:

Politeness score for the third measurement

**‘S_mean’**:

Mean sentiment score, i.e. (S1+S2+S3) / 3

**‘P_mean’**:

Mean politeness score, i.e. (P1+P2+P3) / 3

Example of a Stata command using the file uploaded to OSF:

Stata command to determine the gender distribution of first and last authors

tab gender_first if review==1

tab gender_last if review==1
