# Supplementary material for: Use of ChatGPT to Explore Gender and Geographic Disparities in Scientific Peer Review
Source: J Med Internet Res. 2024 Dec 9;26:e57667. doi: 10.2196/57667 (PMC11667125; doi:10.2196/57667)
Supplement: Multimedia Appendix 3 [file jmir_v26i1e57667_app3.docx]

| Variable | Number of articles, n/N (%) | Number of reviews, n/N (%) | Sentiment score, median (IQR) | Crude p-value^1^ | Adjusted p-value^2^ | Politeness score, median (IQR) | Crude p-value^1^ | Adjusted p-value^2^ |
| --- | --- | --- | --- | --- | --- | --- | --- | --- |
| First authors’ gender |  |  |  | 0.74 | 0.53 |  | 0.53 | 0.74 |
| Female | 42/96 (43.8) | 104/240 (43.3) | 57 (37.5) |  |  | 63 (32) |  |  |
| Male | 54/96 (56.2) | 136/240 (56.7) | 55 (45.5) |  |  | 62 (31.5) |  |  |
| Last authors’ gender |  |  |  | 0.16 | 0.36 |  | 0.69 | 0.71 |
| Female | 30/96 (31.3) | 75/240 (31.3) | 47 (40) |  |  | 62 (26) |  |  |
| Male | 66/96 (68.7) | 165/240 (68.7) | 57 (43) |  |  | 63 (30) |  |  |
| First authors’ affiliation |  |  |  | 0.002 | 0.02^3^ |  | <0.001 | 0.02^4^ |
| North America, Europe, Pacific | 70/96 (72.9) | 169/240 (70.4) | 57 (40) |  |  | 63 (28) |  |  |
| Asia | 16/96 (16.7) | 43/240 (17.9) | 62 (30) |  |  | 65 (23) |  |  |
| Middle East, Latin America, Africa | 10/96 (10.4) | 28/240 (11.7) | 27 (58) |  |  | 43.5 (39.5) |  |  |

^1^ Wilcoxon rank-sum test (for gender) and Kruskal-Wallis equality-of-populations rank test (for affiliation)

^2^ Multivariable negative binomial regression, adjusted for journal, affiliation, and intra-cluster correlation within articles (for first/last authors’ gender), and adjusted for journal and intra-cluster correlation within articles (for first authors’ affiliation)

^3^ IRR Asia vs. Middle East, Latin America, Africa: 1.27 (95%CI 1.06-1.51), North America, Europe, Pacific, vs. Middle East, Latin America, Africa: 1.23 (95%CI 1.02-1.47)

^4^ IRR Asia vs. Middle East, Latin America, Africa: 1.30 (95%CI 1.07-1.57), North America, Europe, Pacific, vs. Middle East, Latin America, Africa: 1.27 (95%CI 1.04-1.54)
